# Insights into significance of combined inhibition of MEK and m-TOR signalling output in *KRAS* mutant non-small-cell lung cancer

**DOI:** 10.1038/bjc.2016.220

**Published:** 2016-07-21

**Authors:** Sophie Broutin, Adam Stewart, Parames Thavasu, Angelo Paci, Jean-Michel Bidart, Udai Banerji

**Affiliations:** 1 Gustave Roussy, Université Paris-Saclay, Département de Biologie et Pathologie Médicales, Villejuif, F-94805, France; 2 The Institute of Cancer Research, Clinical Pharmacology and Trials Team, London, SM2 5PT, UK

**Keywords:** drug combinations, NSCLC, KRAS mutant, MEK inhibitor, trametinib, m-TOR inhibitor, AZD2014

## Abstract

**Background:**

We aimed to understand the dependence of MEK and m-TOR inhibition in *EGFR*^WT^/*ALK*^non-rearranged^ NSCLC cell lines.

**Methods:**

In a panel of *KRAS*^M^ and *KRAS*^WT^ NSCLC cell lines, we determined growth inhibition (GI) following maximal reduction in p-ERK and p-S6RP caused by trametinib (MEK inhibitor) and AZD2014 (m-TOR inhibitor), respectively.

**Results:**

GI caused by maximal m-TOR inhibition was significantly greater than GI caused by maximal MEK inhibition in the cell line panel (52% *vs* 18%, *P*<10^−4^). There was no significant difference in GI caused by maximal m-TOR compared with maximal m-TOR+MEK inhibition. However, GI caused by the combination was significantly greater in the *KRAS*^*M*^ cell lines (79% *vs* 61%, *P*=0.017).

**Conclusions:**

m-TOR inhibition was more critical to GI than MEK inhibition in *EGFR*^WT^/*ALK*^non-rearranged^ NSCLC cells. The combination of MEK and m-TOR inhibition was most effective in *KRAS*^*M*^ cells.

Lung cancer is the leading cause of death in the world. The advent of personalised medicine has seen the introduction of a number of targeted treatments for the adenocarcinoma subset of NSCLC (NSCLC-adeno). Although EGFR (Maemondo *et al*, 2010) and ALK (Solomon *et al*, 2014) inhibitors are established in *EGFR* mutant and *ALK* rearranged lung cancer, there are currently no molecularly targeted agents licensed for use in the remainder of adenocarcinomas (NSCLC-adeno-*EGFR*^WT^/*ALK*^non-rearranged^). There have been recent advances in the treatment of NSCLC-adeno with the introduction of immune checkpoint inhibitors. However, response rates and median overall survival are low with 19% and 12.2 months, respectively (Borghaei *et al*, 2015). Thus, finding new treatments for NSCLC-adeno is an area of unmet need.

We have focused our efforts on the subgroup of NSCLC-adeno-*EGFR*^WT^/*ALK*^non-rearranged^. There are multiple activating mutations in this subset (*KRAS, BRAF, MET, HER-2* and *STK11*), and gene fusions of which *KRAS* mutations form the largest group (Cancer Genome Atlas Research Network, 2014). We tested inhibitors of MEK and m-TOR that target nodes that are downstream of many of these activating events. We studied on a cell line panel the dependence on MEK or m-TOR signalling. We used trametinib, a FDA- and EMA-approved drug (Infante *et al*, 2012), and AZD2014, a drug under clinical investigation (Basu *et al*, 2015), MEK and m-TOR inhibitors, which are known to cause robust pharmacodynamics suppression of signals.

The specific aims of the study were to investigate growth inhibition (GI) following maximal inhibition of MEK or m-TOR in NSCLC-adeno-*EGFR*^WT^/*ALK*^non-rearranged^ cells and to identify any differences between *KRAS*^M^ and *KRAS*^WT^ cell lines. We then studied the effect of additive GI by inhibiting signalling through both MEK and m-TOR nodes compared with GI caused by inhibiting MEK or m-TOR node.

## Materials and methods

### Cell lines and drugs

A panel of six NSCLC cell lines, including three *KRAS*^M^ (A549, Calu6 and H23) and three *KRAS*^WT^ (H522, H1838 and H1651) cell lines, were purchased from ATCC-LGC Standards (Teddington, UK). Trametinib and AZD2014 were sourced from Selleckchem (Munich, Germany).

### Quantification of inhibition of signalling

Maximal reduction of p-ERK1/2 (Thr202/Tyr204; Thr185/Tyr187) and p-S6RP (Ser235/236) was determined by exposing the cell lines to increasing concentrations of Trametinib and AZD2014 over 24 h and quantifying p-ERK1/2 and p-S6RP by ELISA (MesoScale Discovery, Rockville, MD, USA; kit K151DWD; K150DFD) following manufacturer's instructions. Experiments were conducted in triplicates.

### Growth inhibition

Cell lines were exposed to concentrations of Trametinib and AZD2014 shown to maximally inhibit MEK and m-TOR signalling, respectively, for 72 h. The effects of inhibiting these nodes alone or in combination on cell growth were studied using a WST-1 assay (Roche Diagnostics, Burgess Hill, UK). Experiments were carried out in triplicates.

### Statistics

Data are reported as means±s.d. Statistical significance was evaluated by non-parametric Mann–Whitney's tests. Results were subjected to statistical analysis by using the GraphPad Prism software package, v6.01 (GraphPad Software, La Jolla, CA, USA).

## Results

### Determination of Trametinib and AZD2014 concentrations inducing maximal inhibition of signal transduction

**Figure 1:**
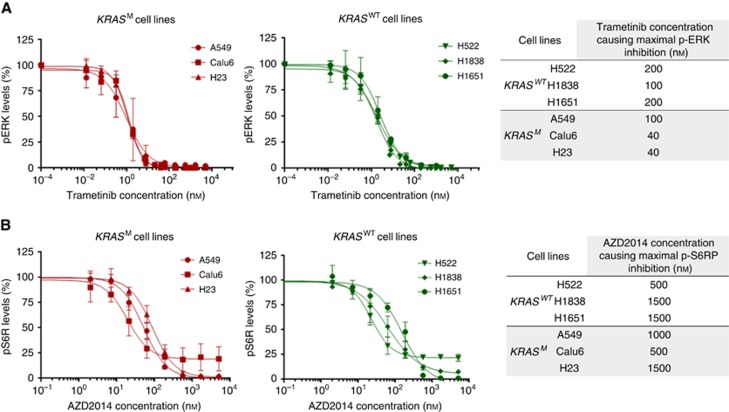
**Determination of degrees of signalling inhibition caused by MEK and m-TOR inhibitors in KRAS^M^ and KRAS^WT^ cell lines.** (**A**) Trametinib concentrations needed to maximally reduce p-ERK levels (table). (**B**) AZD2014 concentrations needed to maximally reduce p-S6RP levels (table). The error bars represent s.d.

NSCLC-adeno-*EGFR*^WT^/*ALK*^non-rearranged^ cell lines were exposed to increasing concentrations of Trametinib and AZD2014 over 24 h and the concentrations of Trametinib and AZD2014 to cause maximal reduction in levels of p-ERK and p-S6RP were determined (Figure 1).

### Determination of dependence of MEK and m-TOR signalling in KRAS^M^ and KRAS^WT^ cell lines

**Figure 2:**
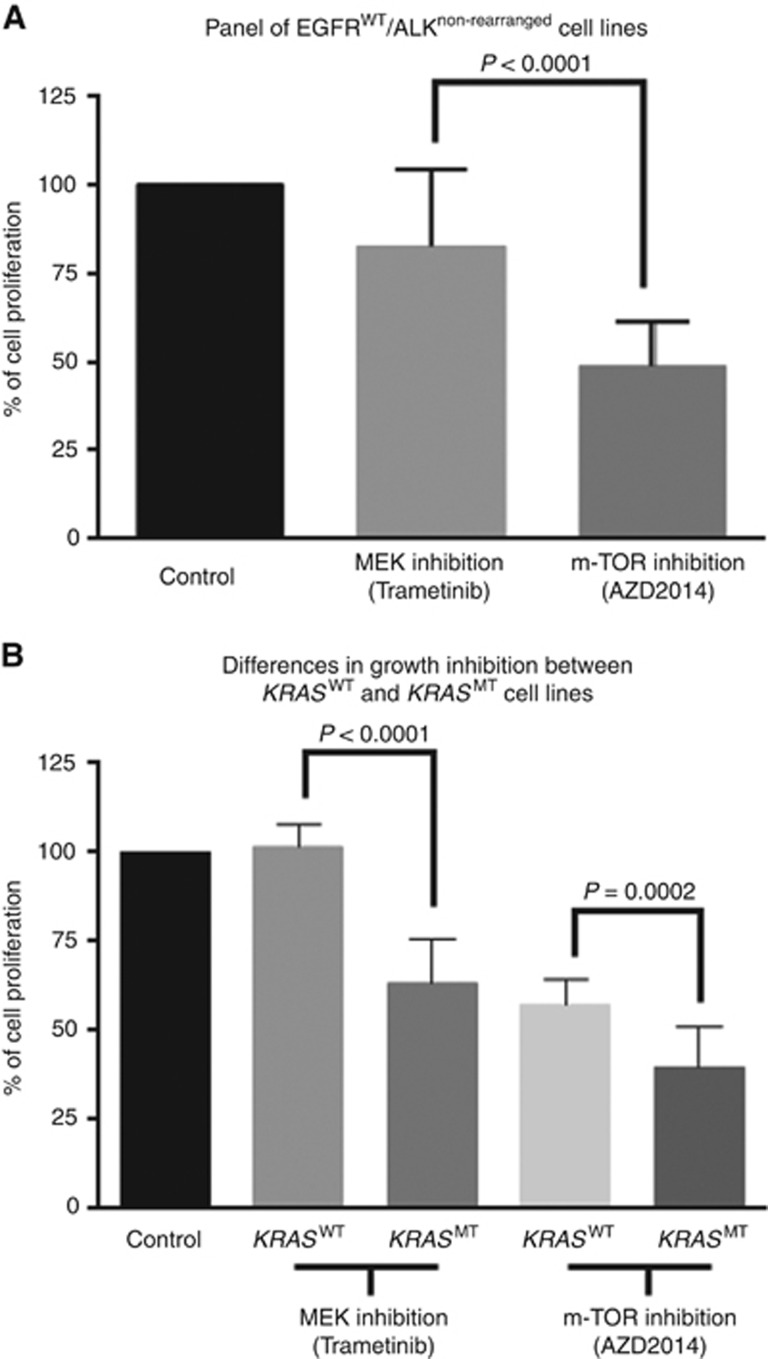
**Growth inhibition upon maximal inhibition of MEK and m-TOR signalling.**(**A**) Growth inhibition in the cell lines panel. (**B**) Growth inhibition in *KRAS*^M^ and *KRAS*^WT^ cell lines. The error bars represent s.d.

Using the concentrations previously determined for each cell line, the mean GI caused by maximal MEK and m-TOR inhibition was 18%(SD22) *vs* 52%(SD13), *P*<0.0001, respectively, in all cell lines (Figure 2A). These data suggest that in this panel, the cells were more dependent on growth on m-TOR signalling compared with MEK. This result is confirmed in *KRAS*^M^ and *KRAS*^WT^ cell lines with GI of 0%(SD6) *vs* 36%(SD12), *P*<0.0001 and 43%(SD7) *vs* 61%(SD11), *P*=0.0002, respectively (Figure 2B).

### Combination of MEK and m-TOR signalling

**Figure 3:**
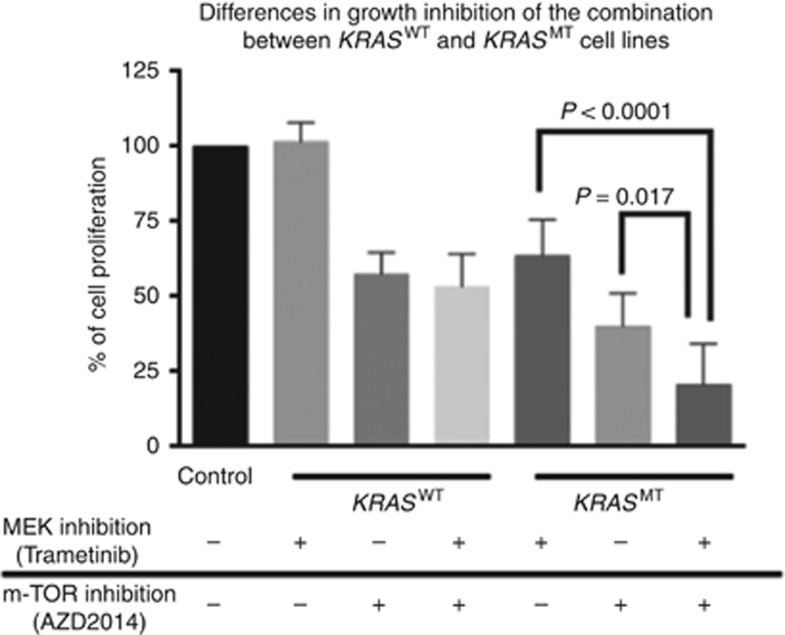
**Growth inhibition caused by MEK and m-TOR inhibitors alone and in combination in *KRAS*^M^ and *KRAS*^WT^ cell lines.**The error bars represent s.d.

As the GI caused by MEK and m-TOR inhibitions were modest, combinations of these were studied, using the same concentrations. Across the cell line panel, the difference between mean GI caused by maximal m-TOR inhibition (52%, SD13) when compared with GI caused by maximal m-TOR+MEK inhibition (64%, SD21) was not significant (*P*=0.089). However, there was a significant difference of GI caused by inhibition of MEK signalling alone, compared with the combination of maximal MEK+m-TOR signalling inhibition, 18%(SD22) *vs* 64%(SD21), *P*<0.0001 (data not shown). Importantly, in *KRAS*^M^ cell lines, inhibition of m-TOR+MEK signalling caused significantly greater GI than inhibition of m-TOR or MEK signalling alone, 79%(SD14) *vs* 61%(SD11), *P*=0.017 and 79%(SD14) *vs* 36(SD12), *P*<0.0001 (Figure 3).

## Discussion

We have used an approach of titrating inhibition of signalling output to decide upon concentrations of drugs to be used in GI experiments as we believe that this approach better reflects the dependence of cancer cell growth to different signalling networks and reduces the reporting predominantly off-target effects of drugs (Stewart *et al*, 2015).

In the NSCLC cell lines studied, we have shown for the first time that maximal inhibition of m-TOR caused significantly more GI compared with maximal MEK inhibition. This is interesting as most efforts of clinically developing signal transduction inhibitors in NSCLC-adeno-*EGFR*^WT^/*ALK*^non-rearranged^ cancers is focussed on using MEK inhibitors. In clinical practice MEK inhibitors response, when used alone, is confined to *KRAS*^M^ tumours, where response rates typically range between 0 and 15% (Zimmer *et al*, 2014; Blumenschein *et al*, 2015). Our data showed that upon maximal MEK inhibition, *KRAS*^M^ cell lines showed significantly greater GI compared with *KRAS*^WT^ cell lines. However, the GI caused by MEK inhibition in *KRAS*^M^ cell lines was modest (36%). This observation is in line with previously published literature (Pratilas *et al*, 2008). In the panel studied, our data suggest that inhibition of MEK signalling has very little effect on growth in *KRAS*^WT^ cell lines within the group of *EGFR*^WT^*/ALK*^non-rearranged^ cell lines.

There have been few single agent studies of m-TOR inhibitors in lung cancer and with very low response rates of 0–5% (Soria *et al*, 2009; Reungwetwattana *et al*, 2012). This discrepancy between the clinical outcomes following m-TOR inhibitor treatment and our data showing that NSCLC cells are more sensitive to m-TOR inhibitors compared with MEK inhibitors, may be explained by signalling though feedback loops and parallel signal transduction pathways (Al-Lazikani *et al*, 2012). Main m-TOR inhibitors evaluated in clinic so far are mTORC1 inhibitors, known to induce feedback loops leading to mTORC2–IRS-1-mediated hyperactivation of PI3K–AKT (Jokinen and Koivunen, 2015), while AZD2014 is a potent inhibitor of mTORC1 and mTORC2 preventing this feedback.

Preclinical studies have suggested combinations of m-TOR and MEK inhibitors in *KRAS*^M^ lung cancers (Engelman *et al*, 2008) but also in N- and HRAS mutant tumours (Kiessling *et al*, 2015). However, these attempted combinations have proven challenging to deliver due to diarrhoea, skin rash and fatigue (Tolcher *et al*, 2015a). Intermittent dosing could be used to reduce the degree of toxicity (Yap *et al*, 2013). We have shown for the first time that inhibition of m-TOR contributes to the majority of the GI in this combination. As our results suggest, in the event of toxicity, clinical trial designs using intermittent dosing should prioritise reducing the dose/frequency of the MEK signalling rather than m-TOR signalling. MEK inhibitors have been used in combination with PI3K and AKT inhibitors which are proximal nodes in the canonical PI3K–AKT–m-TOR pathway and such treatment regimens have shown early promise in the treatment of *KRAS*^*M*^ NSCLC (Shimizu *et al*, 2012; Tolcher *et al*, 2015b). The policy of prioritising inhibition of m-TOR over MEK signalling within the combination may be broadly applicable in across MEK combinations with AKT and PI3K inhibitors but this needs to be validated in those settings.

The combinations of MEK inhibitors and m-TOR inhibitors or more broadly PI3K pathway inhibitors should be evaluated in *KRAS*^M^ NSCLC. Toxicity limits such combinations and intermittent schedules are the only way to deliver drugs at doses that are pharmacodynamically active. In case of toxicity, our data suggest dose interruptions/reductions should be considered in inhibitors of MEK signalling rather than inhibitors of m-TOR signalling. This approach will help refine doses/schedules of MEK and PI3K pathway inhibitors to provide new treatment paradigms for *KRAS*^M^ NSCLC.
